# Safety and Health through Integrated, Facilitated Teams (SHIFT): stepped-wedge protocol for prospective, mixed-methods evaluation of the Healthy Workplace Participatory Program

**DOI:** 10.1186/s12889-020-09551-2

**Published:** 2020-09-29

**Authors:** Laura Punnett, Suzanne Nobrega, Yuan Zhang, Serena Rice, Rebecca Gore, Alicia Kurowski, SHIFT Project Research Team

**Affiliations:** 1 Center for the Promotion of Health in the New England Workplace (CPH-NEW), Lowell, MA USA; 2 Department of Biomedical Engineering, University of Massachusetts Lowell, 1 University Avenue, Lowell, MA 01854 USA; 3 Solomont School of Nursing, University of Massachusetts Lowell, 1 University Avenue, Lowell, MA 01854 USA

**Keywords:** Healthcare workers, Intervention, Mixed methods, Occupational health, Occupational safety, Participatory methods, Total worker health, Worker participation

## Abstract

**Background:**

Healthcare facilities are notorious for occupational health and safety problems. Multi-level interventions are needed to address interacting exposures and their overlapping origins in work organization features. Worker participation in problem identification and resolution is essential. This study evaluates the CPH-NEW Healthy Workplace Participatory Program (HWPP), a *Total Worker Health*® protocol to develop effective employee teams for worker safety, health, and wellbeing.

**Methods:**

Six public sector, unionized healthcare facilities are enrolled, in three pairs, matched by agency. The unit of intervention is a workplace health and safety committee, adapted here to a joint labor-management “Design Team” (DT). The DT conducts root cause analyses, prioritizes problems, identifies feasible interventions in light of the constraints and needs of the specific setting, makes business-case presentations to facility leadership, and assists in evaluation.

Following a stepped-wedge (cross-over) design, one site in each pair is randomly assigned to “immediate intervention” status, receiving the full coached intervention at baseline; in the “lagged intervention” site, coaching begins about half-way through the study. Program effectiveness and cost-effectiveness outcomes are assessed at both organizational (e.g., workers’ compensation claim and absenteeism rates, perceived management support of safety) and individual levels (e.g., self-rated health, sleep quality, leisure-time exercise). Targeted pre-post analyses will also examine specific outcomes appropriate to the topics selected for intervention. Process evaluation outcomes include fidelity of the HWPP intervention, extent of individual DT member activity, expansion of committee scope to include employee well-being, program obstacles and opportunities in each setting, and sustainability (within the available time frame).

**Discussion:**

This study aims for a quantitative evaluation of the HWPP over a time period long enough to accomplish multiple intervention cycles in each facility. The design seeks to achieve comparable study engagement and data quality between groups. We will also assess whether the HWPP might be further improved to meet the needs of U.S. public sector healthcare institutions. Potential challenges include difficulty in pooling data across study sites if Design Teams select different intervention topics, and follow-up periods too short for change to be observed.

**Trial registration:**

ClinicalTrials.gov NCT04251429 (retrospectively registered January 29, 2020), protocol version 1.

## Background and rationale

### Occupational health and safety in the healthcare sector

Healthcare is one of the largest sectors in the U.S. economy, with over 16 million employees, representing 11% of the U.S. labor force [1]. Eighteen of the 30 fastest-growing professions in the U.S. are in healthcare and associated sectors; they are expected to create more than 3.4 million additional jobs by the year 2028 [2]. The largest projected increases are for home health professionals and personal care assistants [2, 3]. Other professions such as nurse practitioners, physician technicians, and medical assistants also represent steady demand as the healthcare sector shifts towards team-based services [3].

Numerous exposures in the healthcare work environment include excessive physical workload (e.g., patient handling), biological agents, cleaning and disinfection chemicals, shiftwork with extended hours, electronic data entry, assaults, and psychosocial stressors. Not all of the resulting health effects are recognized or reported as work-related. Nationally, the reported incidence rate of recordable non-fatal occupational injuries and illnesses for healthcare and social assistance jobs was 4.1 per 100 full-time workers in 2017 and 3.9 in 2018 [4].

The type and severity of injuries vary by job title. Compared to all occupations (private sector), psychiatric aides have among the highest incidence rates for non-fatal workplace injuries requiring days away from work (647.7 per 10,000 full time workers) [5]. Among other healthcare professions, orderlies and nursing assistants’ rates were 283.3 and 255.7, respectively [5]. Rates for registered nurses (RNs) were 88.4, licensed practical and vocational nurses (LPNs) 85.6, healthcare social workers 25.4, and mental health and substance abuse social workers 14.7 [5].

In 2018, incidence rates for nonfatal occupational injuries and illnesses resulting in days away from work among public sector (state government) healthcare occupations were considerably higher than those of their private sector counterparts [6]. Public sector psychiatric aides’ incidence rates were 1475.7 per 10,000 full time workers, nursing assistants 445.9, RNs 194.5, and LPNs 427.7 per 10,000 [7].

The most common events recorded for injuries with days away from work for psychiatric aides were related to violence [5]. In the U.S., violence from patients, visitors, and coworkers is often tolerated as inevitable in the healthcare workplace [8]. The U.S. Occupational Safety and Health Administration (OSHA) has issued prevention guidelines that include engineering controls for primary prevention [9]. However, the typical and often sole strategy offered for preventing health care worker injuries from assault is training in management of patient/client behavior; OSHA’s guidelines are often not implemented and occupational safety personnel are typically not engaged in problem analysis and solutions [10, 11].

Musculoskeletal disorders (MSDs) such as sprains, strains and tears are a common nature of injury among all healthcare workers [12]. For nursing assistants and RNs, overexertion and bodily reaction were the most common types of reported incidents [5]. In 2016, over 19,790 registered nurses in private industry sustained musculoskeletal injuries that resulted in lost work days [13]. In 2015, registered nurses and nursing assistants were two of the top six occupations for lost-workday MSD rates [14]. Nearly 20,000 nursing assistants and over 11,000 registered nurses sustained musculoskeletal injuries that resulted in missed days from work [14]. Other healthcare job groups with notable MSD risk include physical and occupational therapists [15], laboratory technicians [16], housekeeping [17], maintenance workers, laundry, and food service workers [18].

Patient handling is recognized as a major cause of MSDs for clinical staff [19]. For direct care personnel, patient/resident lifting devices can reduce biomechanical load and injury risk [20–24]. However, many facilities do not have sufficient equipment; patient needs and limited co-worker availability for assistance can challenge a structured lifting program. Biomechanical considerations may be complicated by other job features, such as time pressure, staff-supervisor interaction, combative patients [24], and attention to infectious disease risk [25]. Potential effect modification of lifting demands by personal factors such as obesity, cigarette smoking, or depression is still understudied [26]. Further, patient handling is not the only ergonomic demand in caregiving jobs, which also involve lifting of material loads, bending and twisting, and standing for long periods of time.

Job stress and burnout are high among healthcare workers and associated with both psychological and somatic distress [27–29]. Psychosocial stressors, night work, and workplace assault are associated with chronic disease risk factors such as smoking, physical inactivity, obesity, sleep, and mental wellbeing [30–32], health concerns that are not typically attributed to work. Psychosocial stressors have also been linked to high staff turnover intention in nursing homes [32]. Healthcare institutions face substantial challenges in retaining direct care personnel due to workplace health and safety issues. Annual turnover rates for registered nurses are estimated to range from 18 to 26% [33], with cost estimates for each turnover ranging from $62,100–$67,000 [34]. High personnel turnover impacts organizational conditions that increase workplace hazards, creating a vicious cycle and posing challenges for patient safety and quality of care.

This multiplicity of hazards and outcomes poses practical challenges for employee health champions. It can be difficult to prioritize efforts, especially when there is insufficient understanding about how exposures interact with each other, how to interrupt the positive feedback loops, and whether there are common barriers to improving working conditions in these various domains. There is an emerging agreement that workplace health interventions should operate at multiple levels of influence [35], and that health and safety interventions should address work organization and empowering employees in problem identification and resolution [36, 37]. However, there is limited experience with a systematic approach to identify and address the interplay among this myriad of causal factors in order to improve healthcare worker mental and physical health effectively [29, 38, 39].

### Total Worker Health®

The U.S. National Institute for Occupational Safety and Health supports a *Total Worker Health*® (TWH) research program to examine the combined effects of workplace and non-workplace factors on worker safety, health and well-being [40, 41] and to evaluate trials of integrated workplace efforts. Evaluation literature is sparse to date and TWH approaches and goals vary among investigators [42–44].

One strategy to elucidate the relevant occupational and non-occupational risk factors, and how they interact, is participatory action research [45]. In a participatory approach, employees are actively engaged in problem identification and prioritization, decision-making, implementation, and evaluation of the program. This supports effective program implementation and uptake because employees are well qualified to identify opportunities and obstacles present in their work environment [44, 46]. Key features include collaboration, mutual education, and acting on results developed from research questions that are relevant to the community [47, 48]. Previous studies have demonstrated the importance of direct involvement of employees and their levels of influences in the process of program implementation [49, 50]. Decision latitude is itself a key psychosocial determinant of worker health. Thus, providing opportunities for workers to identify problems, set priorities, and design and assess health and safety programs should contribute to improving worker health both directly and indirectly [51].

The Healthy Workplace Participatory Program (HWPP) (http://www.uml.edu/Research/Centers/CPH-NEW/Healthy-Work-Participatory-Program/) is an innovative, evidence-based protocol to facilitate worker participation in priority-setting, root cause analysis, and development, implementation, and evaluation of integrated solutions to workforce health problems. It promotes employee empowerment and engagement to address a wide range of work environment, work organization, and safety and health issues through organization-level change. New understandings emerge as participants reflect on interventions in light of their first-hand knowledge. Preliminary evaluations have shown high user acceptance and positive process outcomes [44, 52, 53].

### Objective

This study, Safety and Health through Integrated, Facilitated Teams (SHIFT), undertakes a prospective evaluation of the HWPP in the healthcare sector. The overall goal is to increase effectiveness of health and safety committees to undertake root cause analysis, problem-solving, and development of solutions. The study will generate quantitative and qualitative evidence regarding its feasibility and effectiveness as a process for obtaining integrated solutions to challenges for worker health. The primary short-term hypothesis is that the health and safety committees will become more active and engaged. Longer-term goals are that the committees become more effective in implementing measures to improve workforce health and safety. Because all study sites come from the same economic sector, generalizable knowledge may be derived regarding the needs and challenges of public sector healthcare facilities. The findings may also identify needs for any further revisions to the HWPP toolkit.

## Methods

### Setting and population

The study is carried out in six public sector healthcare facilities, one pair from each of three agencies. Four of these are run by the state of Massachusetts Executive Office of Health and Human Services: two Department of Mental Health hospitals and two residential facilities for veterans. The other two are inpatient/outpatient facilities of the U.S. Veterans’ Administration.

These agencies all have pre-existing workforce safety and health infrastructure. The Veterans’ Administration has multiple initiatives addressing employee health and safety on a national scope and has been particularly recognized for the excellence of its safe patient handling program [54, 55]. Various committees address Environment of Care, accident review, safe patient handling, violence prevention, and in some facilities health promotion.

In Massachusetts, every executive-level branch of state government is required to have a Safety and Health Coordinator and joint labor-management health and safety committees [56]. State law also mandates that all state agencies provide employees with safety and health protections at least as protective as federal OSHA requirements. Health and Human Services has been one of the most pro-active state agencies, creating an occupational health and safety infrastructure with committees in every agency and assigning a secretariat-level coordinator. Regional workers’ compensation managers review claims data with the coordinator on a regular basis.

Individual study sites were recruited according to the following criteria:

- Active, high-level interest in a participatory, integrated occupational health program.
- An existing occupational health and safety committee with front-line staff members that meets regularly, has a designated facilitator or chair, and is willing to consider expansion of scope to include worker well-being.
- Openness to high degree of worker participation in development and implementation of solutions.
- Leadership recognition and acceptance of the necessary in-kind personnel time commitment (manage survey logistics, coordinate transmission of administrative data files); openness to broadening the scope of issues over time; and willingness to be oriented to the designed management role.

### Study design

**Fig. 1:**
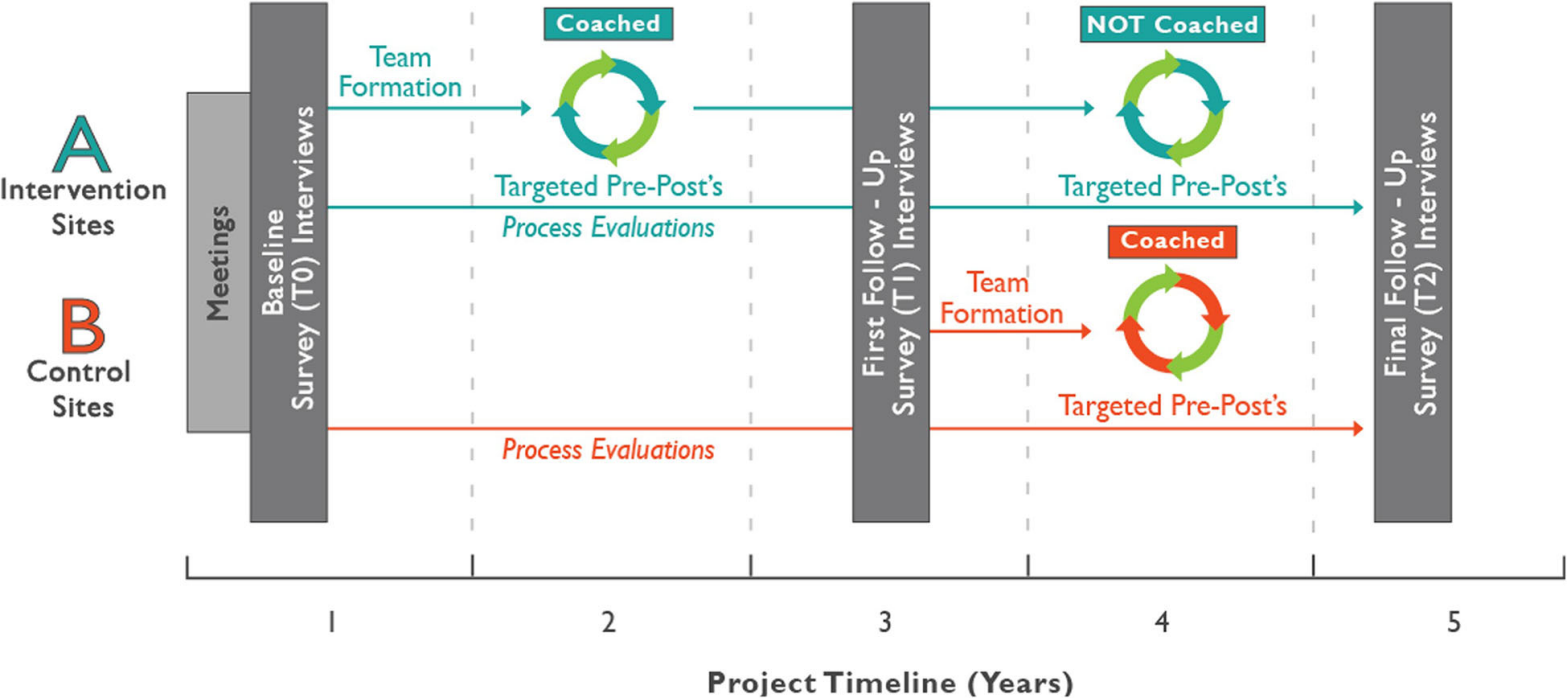
Study timeline for the SHIFT project, with key elements of the stepped-wedge design

The study has a stepped-wedge design [57], meaning a prospective study with concurrent controls that later cross over to intervention (Fig. 1). Study sites are enrolled in matched pairs. Within each pair, one facility is randomly assigned to “immediate intervention” status and receives the intervention as early as possible during the project period, following baseline data collection. The research team provides coaching to co-facilitators and champions throughout the implementation process, in order to enhance fidelity, engagement, and effectiveness. The other facility, designated as the “lagged intervention” site, has status-quo control group status for the first half of the project, continuing with the safety process already in place at that facility until mid-way through the timeline. At that point, the three immediate intervention sites will continue with the HWPP process independently, and the HWPP coaching will be transferred to the lagged intervention sites.

The lag period seeks to obtain adequate observation time with concurrent controls for the intervention sites, as well as follow-up time to examine the interventions that the control sites undertake before the end of the study. During the lag period, all sites have access to health and safety information upon request from the researchers, on topics prioritized by committee members (e.g., ergonomics/safe patient handling; assault prevention; shift work and sleep quality; job stress prevention).

At the time of randomization, findings are presented to the facility Steering Committee (SC) and the Design Team (DT) co-facilitators from the baseline formative assessments (below) of key challenges to employee health, safety, and wellbeing, as well as opportunities and barriers for the participatory process. Similarly, throughout the project the researchers will report key descriptive findings from administrative data and employee surveys (see below) to the DT’s and SC’s for their use in priority-setting.

### Intervention protocol

The HWPP intervention is delivered at the institutional level. A coach from the research team assists each facility to implement the CPH-NEW Intervention Design and Analysis Scorecard (IDEAS) process [53]. The steps in the HWPP program are described and illustrated with training videos and example worksheets on the CPH-NEW website (www.uml.edu/cphnewtoolkit/IDEAS).

The key working group is the Design Team. Because each participating healthcare facility had at least one existing joint management-labor health and safety team, most typically an Environment of Care committee, the HWPP has been adapted to this membership structure. During the recruitment and start-up period, specific instructions are provided regarding equal numbers of DT members from the rank-and-file workforce and management, and avoidance of direct supervisory relationships between any pair of DT members. The researchers expended substantial time in pre-study communication with facility leadership as well as the multiple bargaining units at each site to ensure clear understanding of these ground rules and selection of representatives enthusiastic to participate. Two employees per site were trained as co-facilitators, one from labor and one from management. Besides seeking balance in perspectives, having co-facilitators seeks to ensure continuity in case of employee turnover and to set the stage for longer-term sustainability.

Within each facility, the existing management decision-making group is designated the HWPP Steering Committee and a senior manager plays the role of program “champion.” The champion attends the initial trainings, participates in quarterly study oversight meetings, and agrees to maintain regular communication with the DT co-facilitators. A structured process defines the sequence of activities and key communications between the DT and SC, which provides the DT with constructive feedback about the proposals, selects interventions to be implemented, and provides any needed resources for refining, implementing, and evaluating interventions.

The researchers orient all DT members and the champion to the study design, the HWPP, and the study roles. Additional training is provided as the DT moves through the stages of the HWPP: meeting management skills (agendas, minutes, tracking action items); use of fishbone diagrams to identify root causes of selected safety/health concerns; development of solutions through systems analysis; strategies for presenting these to the facility leadership team; and techniques to evaluate DT activities and use these results to guide refinements and related solutions. Finally, the teams will present these ideas to leadership, creating effective and meaningful dialogue.

Because interventions are designed through a participatory process, the DT and SC members at each site – not the researchers – select the intervention goals and activities to be carried out. Based on prior experience in the healthcare sector, review of earlier workers’ compensation claims from these three agencies, and conversations during the recruitment process, key issues likely to be selected at the participating facilities include psychosocial job stress and burnout; verbal and physical assault; physical ergonomics (including but not limited to patient handling); overtime, shiftwork, and sleep quality; and traditional safety issues (e.g., slips, trips and falls). In accordance with the Total Worker Health mission, the DT’s are specifically invited to consider interactions between the work environment and personal well-being/health behaviors (exercise, nutrition, sleep, etc.), and whether these might have shared or overlapping solutions.

The researchers anticipate at least one issue to be selected and intervened on per year at each site, once active coaching begins. By the mid-point of the study, each immediate intervention DT should have completed at least two complete IDEAS cycles and be prepared to continue the process more independently. The coach will reduce active assistance while maintaining contact through regular telephone or e-mail check-ins with DT members and co-facilitators to assess progress, learn about problems or concerns, and offer advice if requested. Research staff will continue to be available as a technical resource when DTs take up new topics.

### Evaluation

**Fig. 2:**
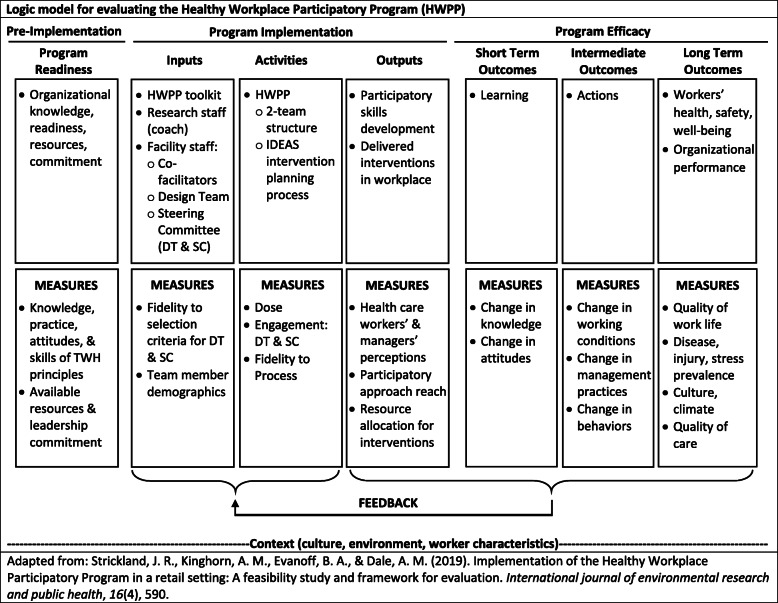
Logic model for the Healthy Workplace Participatory Program

The program implementation process and effectiveness will be evaluated, using qualitative and quantitative methods, at the levels of the institution, program team members, and individuals affected by interventions generated by the teams (Table 1). A program logic model of the HWPP outputs and outcomes guides the selection, development and use of the evaluation elements (Fig. 2), informed by that of Strickland et al. [58]. This section describes process evaluation measures to evaluate HWPP outputs, and formative and effectiveness evaluation measures to describe HWPP outcomes.

**Table 1:** Qualitative and quantitative instruments for process, formative, and effectiveness evaluation in the SHIFT project

Instrument	Description	Content	Administration Frequency
**Process Evaluation**			
*Mobile App, “HWPP Assistant”*	Meeting assessments and time logs by DT and SC members	• DT members and co-facilitators: participation^a^, knowledge, satisfaction, team effectiveness, engagement^a^, value • DT co-facilitators: confidence in facilitating; quality and usability of HWPP materials • SC members: awareness of team developments, organizational disturbances, team effectiveness, value, engagement • Log of project-related time (level of participation^a^)	After each meeting
*Coach Notes; Research Assistant Notes*	Systematic, qualitative observations of DT, SC, and joint DT/SC meetings	• Attendance^a^ • Team dynamics^a^ • Quality of Design Team facilitation (DT only) • Level of management support (DT only) • Progress through the structured IDEAS intervention design process^a^ • Any difficulties with the IDEAS process; any adaptations made	After each meeting
*Design Team Meeting Records*	Person-time invested; Fidelity of DT meetings to HWPP protocol	• DT meeting dates^a^ • Time spent in meetings^a^ • Number of attendees^a^ • Agenda items covered from the protocol	After each meeting
**Formative and Effectiveness Evaluation: Short and Medium-term Outcomes**			
*IDEAS Worksheets*	DT work record of progress on IDEAS steps (summary worksheet)	• Content of discussions and interventions at each step: knowledge, breadth of topics covered over time^a^	After IDEAS Step 5
*Process Check-up*	DT and SC members: periodic survey evaluating the HWPP	• Organizational support and engagement^a^ • DT and SC engagement^a^ • Program facilitation • Intervention planning/implementation	After IDEAS Step 5, then every 6 months during coaching
*Design Team Survey*	DT survey on members’ attitudes and perceptions of HWPP	• Member engagement^a^; Involvement^a^ • Managerial support for DT interventions • Shared DT/SC knowledge and understanding about root causes of safety and health concerns • Quality of organizational communication • Expected HWPP impact • Any unplanned consequences observed (negative or positive)	After IDEAS Step 5, then every 6 months during coaching
*Interim Champion & Facilitator Interviews*	Semi-structured interviews for champions and co-facilitators	• Satisfaction regarding program implementation • Quality of and trends in DT and SC effectiveness, engagement^a^, involvement^a^, communication and organizational support • Expectations regarding impacts of the participatory program overall and from the interventions being designed and implemented by the teams	Every 6 months during coaching
**Formative and Effectiveness Evaluation: Longer-term Outcomes**			
*All-Employee Survey*	Self-administered survey (paper and pencil) for all facility employees	• Self-rated health, body weight, health behaviors, perceived health needs, attitudes and beliefs • Symptoms of burnout and depression, history of injury and chronic disease • Location/severity of musculoskeletal symptoms, related functional impairment • Perceptions of working conditions, physical and psychological job features, organizational culture and climate • Knowledge of and perceived effectiveness of health and safety programs	Baseline & study end
*Organizational Readiness Survey*	SC members, DT co-facilitators, and union leaders: organization’s readiness to form participatory DTs and resources available to support the HWPP (45-item survey)	• Existing programs for safety, health, and well-being • Current program approaches for safety, health, and well-being • Current resources available for safety, health and wellbeing • Resources and readiness for change initiatives to improve safety, health and wellbeing • Resources and readiness for use of teams • Teamwork in work groups • Resources and readiness for employee participation • Management communication about safety, health, and well-being	Baseline & study end
*Leadership Interviews*	Scripted, open-ended interviews for SC members, DT co-facilitators, union leaders	• Perceived workforce safety and health concerns • Employee involvement^a^ in, successes of, and challenges to the existing safety program • Current strengths, opportunities, and obstacles to the HWPP within the facility • Shared DT/SC knowledge and understanding about root causes of safety and health concerns • Expected HWPP impact • Any unplanned consequences observed (negative or positive)	Baseline & study end
*Pre-Post Intervention Surveys*	DT-administered surveys	• Topic-specific measures based on key performance indicators (e.g., reduction of workplace exposures, short-term changes in behavior). Workforce sample determined by scope of intervention.	Pre- and post-IDEAS interventions

^a^primary outcome

#### Process evaluation

The HWPP relies on continuous participatory engagement of program participants and the workforce so that the workers’ own knowledge can inform customization of the program to local conditions, in keeping with the principles of participatory action research. Thus, a substantial amount of planned evaluation addresses the implementation process and corresponds to the Logic Model elements of “Activities and Outputs.”

Process evaluation addresses potential generalizability of the results by documenting specific conditions that necessitate customization as well as quantifying personnel and other resources needed for implementation. The choice of process measures is informed by implementation science research frameworks [59–61] with adaptations for the occupational safety and health setting [62]. For example, the level of participants’ engagement, enthusiasm and confidence in their roles is expected to predict the success of the program. Quality and usability of the program materials are also likely predictors. Characteristics of the implementation process include the participants’ efforts in planning, engaging, executing, reflecting, and evaluating the program steps. External forces and organizational characteristics of the study sites may either facilitate or impede program implementation.

Process measures registered as *primary outcomes for this clinical trial* are team attendance, team engagement, level of committee activity, and scope of health, safety, and well-being issues addressed. Process measures registered as *secondary outcomes* include proportion of the HWPP protocol and content followed (fidelity), and development of shared labor-management awareness of job-related health issues.

#### Formative evaluation

Pre-program data on workforce health, safety, and well-being provide baseline assessments of potential problems to be addressed. Workers’ compensation (WC) and administrative data are collected retrospectively at baseline to provide context and understanding of major health and safety needs. Each site will provide de-identified electronic administrative data files annually covering workforce size and demographics; employee retention by job group; workers’ health-related absenteeism, and WC claims and costs. These data will also be collected prospectively over the course of the study.

The baseline All-Employee Survey (AES) assesses the starting conditions for individual-level employee health status, burnout, and work ability; frequency of threats, assault, and harassment of employees; staff-reported quality of care of patients/residents; and perceived organizational support for workforce health, safety, and wellbeing.

Baseline interviews with upper and mid-level managers, as well as union leaders, assess perceptions of priority employee safety and health concerns, alignment of the HWPP with organizational goals, as well as expectancies for program outcomes and sustainability. (The interview guide was developed for this study (see Supplementary File). Organizational readiness surveys of the same organizational leaders assess perception in organizational resources and readiness for safety and health programs, teamwork and communication, and organizational-level readiness for worker participation in program design. These domains are associated with successful implementation of participatory ergonomics programs and safety-related organization change efforts [63]. Baseline data from organizational leaders will identify training needs and other possible resource gaps to address for successful implementation of the HWPP. Follow-up data will be gathered through regular interviews and surveys of the same managers, program champions, and union leaders, as well as DT members.

#### Effectiveness evaluation

Effectiveness outcome measures include changes in the formative measures over time, designated in the Logic Model as “Short Term,” “Intermediate,” and “Long Term” outcomes (Fig. 2). Short-term outcomes include changes in knowledge and skills of Design Team members (e.g. greater awareness of perspectives of other employees in different work units and at different levels of the organizational hierarchy, increased quality of communication and level of engagement, and focus on safety, health, and well-being concerns of employees), as well as management commitment of resources for implementing safety/well-being interventions.

At the facility level, the goal is that locally-designed interventions on DT-selected topics will be implemented through the authority of the Steering Committee. Their eventual effectiveness on relevant metrics of organizational performance will be assessed at multiple levels. We expect the training provided to teams to result in the production of intervention ideas that reflect both work and non-work contributors. Therefore, the team-generated interventions will be assessed for TWH characteristics.

Organizational performance will be assessed through interim interviews of champions, co-facilitators, and organizational leaders to assess expectancies related to implementation and impact of interventions selected by the Design Team. A survey will be administered to DT members semi-annually to collect similar measures, plus measures of personal involvement, program value, and management support.

Longer-term effectiveness analyses will utilize data from the AES, administered at least twice during the study. Outcomes of interest include changes in workers’ exposures, stressors, work climate, and perceived organizational support, as well as individual-level health, safety, and wellbeing (Table 1). Organizational readiness surveys will be re-administered with organizational leaders to assess changes in resources and readiness for participation, change initiatives, safety and health teamwork and communication.

While valuable for tracking large trends in a workforce, the AES may not be sensitive to specific interventions, especially if these target certain units or workforce sub-groups. The IDEAS toolkit calls for the DT to identify measurable key performance indicators for each intervention. The DT will use these to generate topic-specific measures such as pre/post “mini-surveys” to assess impact in the appropriate segment(s) of the workforce. The HWPP intervention includes investigator coaching on how to design and conduct targeted pre/post surveys. Thus, carrying out these targeted surveys will itself be recorded as a measure of committee effectiveness. If different DT’s select the same issue during the study, the investigators will suggest use of overlapping measures, to facilitate cross-facility comparison and pooling of data on effectiveness.

### Data analysis

#### Process evaluation

Short-term process measures will be summarized for each committee over time and compared between the control and intervention groups. For example, changes in implementation metrics will quantify the extent to which the participatory DTs utilized the HWPP program (uptake) and adhered to it (fidelity, sustainability). The DT surveys and Coach notes will be summarized to document the development of team functionality, independence, ability to plan and implement activities, and ability to evaluate success or setbacks of activities to improve the process in the future. We will also describe obstacles and opportunities in each setting, and the extent to which these are similar among agencies.

We will summarize the accomplishments of the HWPP and the factors observed to affect implementation and outcomes of the program; the challenges and successes of long-term team maintenance; and how these were different from or similar to the challenges and successes of the health and safety committees in the control sites before the lagged interventions. We will discuss the implications of these combined qualitative and quantitative findings for potential long-term sustainability of the participatory programs. These findings may be relevant to post-hoc analyses of any site differences in effectiveness and will also inform future refinements in HWPP content or delivery.

#### Formative evaluation

Initial analyses of individual-level AES data will quantify the baseline magnitude of worker health and health-related indicators, such as burnout/depression, musculoskeletal symptoms and function, recent assault, and acute injury. Data will be summarized by study group (immediate/lagged intervention), facility, and job group. Cross-sectional associations with work environment features will focus on dependent variables prioritized by DT and SC members in the initial surveys and interviews. Candidate job characteristics are physical and psychosocial job demands, decision latitude, perceived supervisor and coworker support, and work schedule.

We will also generate descriptive data on institutional characteristics such as average ratings of organizational culture and climate; quality of safe patient/resident handling and other worker health or safety programs; compensation claim rates; employee turnover rates; and clinical staffing ratios. These analyses will be done by site for customized reports, and by agency type and study group to assess potential confounding and effect modification.

#### Effectiveness evaluation

The pre/post “mini-surveys” will be analyzed to assess short-term effectiveness of locally-designed interventions within the proportion of the workforce expected to benefit. Employee participation in DT-promoted activities will be compared between study groups and over time (pre- vs post-intervention).

Organizational-level improvements in worker engagement, communication or shared understanding between management and workforce, SC authorization of resources, or uptake and institutionalization of new practices will be interpreted as effectiveness of the participatory intervention process, at the overall study level.

Assuming that both groups intervene on similar topics over time, pooled effectiveness analyses will compare AES data in the immediate intervention and lagged intervention groups. If sufficient numbers of workers complete more than two surveys, we will also use a difference-in-difference approach. Reporting of individual-level outcomes (e.g., changes in occupational exposures, health beliefs and behaviors) will follow the CONSORT guidelines for cluster randomized trials [64].

Statistical power calculations for intention-to-treat comparisons of several key outcomes (Table 2) were conducted for difference-in-difference analysis, with two-sample differences at the population level. The facilities within each pair are of different workforce sizes, so we assumed conservatively the smaller of the facilities from each pair in both groups. Full-time equivalent denominators were assumed to represent 90% of headcount; a 75% response rate gave samples of about 1650 workers per group. We used a binomial distribution for proportions (e.g., smoking) and Gaussian distribution for means (e.g., body mass index (BMI)). Starting values were taken as those found in our prior healthcare study using a similar instrument [31, 32, 65]. Modest differences should be readily detectable, assuming facility-wide implementation of each HWPP intervention.

**Table 2:** Smallest detectable differences in selected outcomes with 80% power (alpha = 0.05, two-sided *p*-value)

Outcome	Baseline value	Detectable difference
Injury rate	0.71 / FTE	.062 / FTE
Smoking	Prevalence 25.6%	6.0%
BMI	Z = 28, SD = 9	0.9
Health self-efficacy	Z = 27, SD = 8.5	0.8
Work ability	Z = 8.9, SD = 2	0.19

In addition, where interventions have addressed health outcomes that are generally attributed to working conditions, workers’ compensation claims will be compared over time. For example, with an occupational safety and health intervention such as installation of safe patient handling equipment, benefits may be measured through change in WC claim rates and costs for back injuries and muscle strains.

#### Cost-effectiveness evaluation

We will estimate cost-benefit for each intervention and cost-effectiveness (cost-outcome) for selected outcomes. Cost will be represented by organizational inputs (equipment, personnel effort) and benefits in terms of adverse outcomes avoided (e.g., worker injury prevented, dollars of WC claims avoided, unit of body weight lost, resident fall prevented).

The primary challenges to cost-benefit analyses lie in the measurement of costs of intervention and in assigning a monetary value to the outcomes. Data from the control group will be used to estimate the cost (lost workdays, turnover, training, hiring substitutes) of not intervening. The analyses will calculate “net present value,” or the sum of all costs and benefits associated with the intervention, measured at a specific time point by discounting each cost and benefit by the opportunity cost of capital [66]. This sum is positive when an intervention provides a benefit greater than its costs. Some intervention costs involve large initial outlays and others recur over time, such as the costs of equipment maintenance and employee training. Benefits may or may not be immediately visible or may also increase over time. This method allows adjustment for the difference in timing of each monetary value through the discounting of each amount by the opportunity cost. In other words, the benefits of interventions are incremental, estimated per year. This also implies that, in order to be cost-effective, the benefits of intervention need to be large enough to outweigh the opportunity cost of capital that could have alternative uses.

As noted above, for some interventions, changes in WC costs will be meaningful. Other interventions may affect health outcomes not generally attributed to working conditions, such as obesity or sleep quality and its sequelae. In this case, benefits would likely be severely underestimated by relying only on WC costs, and other metrics are needed. In either case, benefits may also include reduced worker fatigue, higher productivity, fewer employee errors, or improved patient care. To aid in understanding, some AES items address knowledge and use of the internal reporting system and WC; self-assessed work ability and productivity; perceived quality of patient care; and intention to leave the job. However, these benefits are difficult to assign a monetary value. We will work with the partnering facilities to attempt to develop conservative estimation algorithms, using survey measures and administrative data.

An approach that does not require assigning dollar value to health or other non-monetary, measurable benefits (improved worker morale) is cost-effectiveness or cost-outcome analysis, which produces estimates of the cost to achieve a given unit of benefit, e.g., reducing body mass index by one point. The cost is estimated by recording the efforts and resources required to implement the program and applying dollar values to each one, as above. We will then estimate the investment relative to the program benefits achieved.

### Protection of human subjects

All subject recruitment is directed by the study personnel. If contact is made through a third party (e.g., employer newsletter), all further communications occur privately. All participants are asked to sign activity-specific informed consent forms explaining the procedure, offering to answer questions, and informing them that they are free to withdraw their consent and discontinue participation in the study at any time without prejudice to their employment situations. Personal identifiers are requested for contact information and to ensure accurate record linkage across stages of data collection. They are not kept with the data files and will be destroyed at the end of the study. Data values for small cells (e.g., fewer than 5 people per facility/job group/gender) will not be reported, to protect anonymity.

## Discussion

Overall, the SHIFT study will extend the knowledge about the effectiveness of a participatory approach to Total Worker Health in the healthcare sector. The HWPP will be implemented with attention to all job groups, for the broad objectives of protecting employee safety and fostering health and well-being. It is hoped that this integrated approach will complement and strengthen existing workplace safety and health initiatives and promote organizational learning, producing synergistic effects [46]. We expect to obtain valuable information on program effectiveness for improving employee safety and health, program impact at the individual and organizational levels, facilitators and barriers to program implementation, and how the HWPP might be further improved, especially for public sector facilities.

### Methodologic considerations

Although workplace integration of programs in occupational health and health promotion seems desirable, there are challenges. One is that integrated programs require more time, effort, and commitment from the organization [67]. For example, we expect the management steering committee to provide timely constructive feedback to interventions proposed by the DT, as well as financial and organizational resources for implementing and evaluating them. The biggest barrier to front-line employee participation in a previous healthcare program was lack of release time for employees [68]. Although obtaining such commitment from the organization takes time and effort, we believe it will be worthwhile because organization culture, environment, and leadership support are closely associated with employee safety and health [32, 69].

The standardized All-Employee Survey will be valuable for tracking large trends in the study population, but if the intervention only benefits a sub-group, any effect could be diluted and difficult to observe. Further, individual health outcomes, as well as workplace culture and organizational features, may evolve gradually enough that there is not adequate time to see changes. We have included a number of proximal endpoints to alleviate this potential challenge. For example, perceived management support of safety relates to longer-term outcomes HWPP, through the multi-level communication and organizational learning that results from the IDEAS process.

Because of the high degree of participatory decision-making, specific health targets selected may vary among DTs. The facilities have generally indicated a similar set of concerns, but even if intervention topics overlap across the sites, they will not necessarily be taken up in the same sequence. This requires that we examine both generic injury/illness outcomes, as well as problem-specific outcomes. Depending on the sequence in which the same topics are addressed in each facility, the length of follow-up time for those endpoints may vary and need to be adjusted for accordingly.

The gold standard for an intervention study is the randomized clinical trial (RCT). Ideally this study would use a cluster randomized design, where groups with similar background risk (on average) are randomly assigned to the intervention or to a control group that receives standard or current services. However, the benefits of randomization to prevent confounding are not realized except with a large sample. In this case, the intervention is administered at the level of the entire facility, and resources do not permit enrollment of enough sites. In any case, it would be difficult to find enough workplaces of a similar nature. The alternative is to compare the treatment groups on baseline characteristics that might influence the outcome. Differences can then be adjusted for with stratified analysis multivariable regression. The double-blinding in RCTs also prevents the possibility of information bias. However, double-blinding is infeasible for organization-level interventions, and other study designs, such as the stepped wedge, maybe be of equal value with attention to reducing bias [70–72]. Further, RCTs have other disadvantages, such as limited capacity to assess multi-dimensional interventions – such as this one - and incompatibility with community trust, choice, and participation often needed for successful program design and evaluation. The stepped-wedge design was selected to achieve comparable study engagement, survey participation, and data quality in all sites.

### Study revisions after the start date

After the study began, one facility withdrew by order of a manager in a central agency office, for administrative reasons. We continue to support the other facility in that pair as an immediate intervention site. Data from the unpaired facility will be utilized in comparisons with the other two immediate intervention sites, to assess generalizability of results and describe program sensitivity to institutional context.

Due to administrative delays and personnel changes, the SHIFT start-up process took about 2 years, whereas the original study design had envisioned less than 1 year needed until the Design Teams began application of the IDEAS Tool. For meaningful evaluation, the coaching in the immediate intervention group should be maintained long enough to complete at least two full intervention cycles. Coaching in the lagged intervention group will then be concurrent with that in the first group. This preserves roughly 1.5 years of the core comparison between concurrent experiences in Groups A and B. In fact, this timeline corresponds closely to the typical stepped-wedge design, where interventions are added progressively to later sites but not necessarily withdrawn from the initial ones.

### Potential impact

This study of healthcare workers is innovative in not being limited to clinical personnel. There are numerous health and safety challenges for workers in other healthcare jobs, especially those of lower socioeconomic status, such as laundry, food service, housekeeping and maintenance [18].

This work will benefit both workers and employers. Rooted in participatory action research with organization support, the HWPP will ideally be sustained after the researchers step back, because employees will benefit from an improved work environment as well as safety and health through interventions proposed and implemented on their willingness. Employer support might be motivated by positive effects such as improved worker morale and engagement and lower rates of compensation claims, missed workdays, and job turnover.

## Supplementary information

**Additional file 1.** Facility Leadership Interview Protocol - SHIFT Project. Interview guide for facility and union leaders.

## Data Availability

Data collection forms are available from the research team at: cphnew@uml.edu. Data files are not publicly available due to privacy guarantees to study participants, many of whom could be identified from their roles and demographic characteristics even in de-identified data sets.

## Abbreviations

AES: All-Employee Survey
CPH-NEW: Center for the Promotion of Health in the New England Workplace
DT: Design Team
HWPP: Healthy Workplace Participatory Program
IDEAS: Intervention Design and Analysis Scorecard
LPN: Licensed practical nurse
MSD: Musculoskeletal disorder
OSHA: Occupational Safety and Health Administration
RN: Registered nurse
SC: Steering Committee
SHIFT: Safety and Health through Integrated, Facilitated Teams
TWH: Total Worker Health
WC: Workers’ compensation
