# Supplementary material for: Safety and Health through Integrated, Facilitated Teams (SHIFT): stepped-wedge protocol for prospective, mixed-methods evaluation of the Healthy Workplace Participatory Program
Source: BMC Public Health. 2020 Sep 29;20:1463. doi: 10.1186/s12889-020-09551-2 (PMC7526105; doi:10.1186/s12889-020-09551-2)
Supplement: Supplementary file 1 — Additional file 1. Facility Leadership Interview Protocol - SHIFT Project. Interview guide for facility and union leaders. [file 12889_2020_9551_MOESM1_ESM.pdf]

Facility Leadership Interview Protocol - SHIFT Project

Note to interviewer—be sure to have a copy of the participant's organizational readiness survey responses printed (one copy for participant and one for interviewer)

Introduction (5 mins): Thank you for taking time for this interview today. My name is (name) and this is (name), who is a Research Assistant. We are here from University of Massachusetts Lowell as part of the SHIFT research study to introduce a new program for health improvement in your workplace. SHIFT means **Safety in Healthcare through Integrated Facilitator Training**.

The new program will integrate the benefits of safety with health promotion in your workplace through a participatory process.

The health and safety committee will take an active role in all phases of the introducing new solutions to safety concerns in your workplace. University of MA Lowell will evaluate the program by collecting data from the safety committee and others at various times, starting with this interview. We'd like to take a few moments to review your rights as a research participant. Then we can begin the interview.

REVIEW INFORMED CONSENT FORM, COLLECT SIGNATURE, COLLECT DEMOGRAPHICS SHEET

Introduction to the SHIFT study (reviews these details with participant)

I'd like to begin by describing the SHIFT study so you are familiar with what will be happening over the next few months and beyond.

(If the participant is already familiar, simply show the fact sheet and quickly review the main study activities and timeline.)

SHOW the SHIFT fact sheets and explain the following points:

- The SHIFT study is a 5-year project to evaluate the effectiveness of a participatory approach to safety, health and well-being.
- The research goal is to assess whether an established toolkit, developed by our research center, can help to **enhance the effectiveness** of the safety committee by **involving more front line staff,** and following a **step-by-step process** to create new safety and health interventions that have a business case.
- You and others will serve as Steering Committee members to oversee the HSC participatory program activities.
- The Health and Safety Committee will act as a Design Team. They will meet monthly. The Design Team members will include all relevant labor groups and will consist of at least 50% of front line staff. The committee will be led by two co-facilitators—labor and management.
- A UMass Lowell research team member will attend the Design Team meetings and will coach the Co-facilitators as they implement the participatory program.
- The Design Team will work on ONE topic at a time. They will learn how to do a root cause analysis of the issue at hand. They will consider a wide range of solutions and develop three intervention alternatives. They assess potential reach, effectiveness, resources, and obstacles to each alternative and present them to the Steering Committee. (Steps 1-5).

- The Steering Committee will receive proposals for the intervention alternatives from the Design Team and will consider which to support. They will retain the authority to select which interventions to approve for implementation. They may collaborate with the Design Team to modify as needed. Once a decision is made about which intervention to adopt, the Steering committee will then be responsible for finding resources to implement interventions and then evaluating them.
- Your facility and a similar facility within your agency have been paired for this study. One of you will receive the intervention right away, and the other will receive the intervention two years from now. We'll toss a coin to determine your assignment. Either way, your facility will participate in data collection activities – surveys, interviews, and monthly process evaluation using a phone app.

Do you have any questions about the SHIFT study before I begin with the prepared interview questions?

Now we can begin the interview.

Interview Questions (45 mins)

Participant information and context

1. We would like to begin today by asking you for some basic information about yourself.

   a. What is your job title?

   b. How long have you worked at this facility?

   c. What is your role (if any) related to employee safety/health/well-being in this facility?

2. Health and Safety Needs of Employees

I'd like to ask for your perspectives about employee safety and health in this organization. We'd like to understand the kinds of issues that are important to different people here.

2.a In your opinion, what are the most important safety and health priorities for employees in this facility? (i.e., the most important issues or hazards)

Now, I'd like to get your thoughts about the work environment here, and how it relates to health for individual employees.

2.b. What aspects of this workplace can make it difficult to stay healthy or engage in healthy activities? Consider the physical environment, the social environment, the way that work is organized, and/or job tasks.

Probes (if needed)

- Are there concerns about workload, work pace, or other demands that can get in the way of health?
- Are there work policies that seem to influence health?

3. Health and Safety Programs

Next, I'd like to ask for your thoughts related to safety and health programs in this facility. We'd like to gain a better understanding of how the organization operates in this area.

For the next few questions, I'd like you to think about the **Safety and Health committee** in this facility.

3.a. What are the main areas of focus for the safety committee? What has the committee been working on?

3.b How does your health and safety committee select priorities for what they work on?

3.c. How does the organization determine if the safety committee is functioning effectively?

- Do they meet regularly? Do they have a focus? How do they organize their work?

Now I'd like you to think about the safety program generally.

3.d. How do senior managers in this organization measure success when it comes to employee safety, health and well-being?

- For instance, what data are collected and monitored?

3.e. What do you see as the strengths of the current safety program?

3.f. What do you see as the challenges or obstacles for the safety program?

3.g. Are there any safety and health concerns that are not yet sufficiently addressed?

4. Review of Organizational Readiness Survey

Review "unclear" questions only from Organizational Readiness Survey

Before this interview, you completed a brief survey of organizational readiness for implementing a participatory, Total Worker Health programs. Because this is a new questionnaire, we are asking survey takers to help determine if the wording on any questions is unclear.

If you checked "unclear" for any of the questions, I'm going to review those questions now, and will see if you would like an opportunity to revise your responses for just for those questions.

Review "unclear" questions on survey printout, ask participant what they thought the question was asking, clarify the intended meaning of the question.
Ask if current response is still correct or if participant wants to revise the response (only for the "unclear" question(s)).

Summary and closing questions
Thank you very much for all the insights you've shared in the discussion so far. Before we close, I'd like you to reflect back to the research activities I described at the start of our conversation.

- The UMass Lowell research team will train the Health and Safety committee to **involve more front line staff,** and follow a **step-by-step process** to create new safety and health interventions that have a business case.
- You and others will serve as Design Team or Steering Committee members to oversee the HSC participatory program activities.

5.a. Based on what you know about the study activities, what do you think are most important outcomes of this study for your organization?

5.b. What do you see as barriers or challenges to the study being successful?

NOTE: IF THERE WERE DIMENSIONS WITH LOW SCORES IN ORG READINESS SURVEY, YOU MIGHT ASK ABOUT CONCERNS RELATED TO THOSE AREAS IF THERE IS TIME.

5.c. Do you see any alignment of this project relative to the values/mission/vision of this organization? If so, how?

5.d. What haven't I asked that you think would be important to know about your organization before we begin the study?

Thank you again for your time and valuable insights. This concludes the interview.

If you would like to contact me to ask questions or to provide additional information please feel free to contact me by phone or email (provide business card).

Use sentence below only if appropriate. Interview participants should receive the organizational readiness survey BEFORE the leadership interview commences.

If you have not already completed an Organizational Readiness Survey we will send you an email with a link to completed it following this interview.
